# 50 Years of Bong-Han Theory and 10 Years of Primo Vascular System

**DOI:** 10.1155/2013/587827

**Published:** 2013-07-31

**Authors:** Kwang-Sup Soh, Kyung A. Kang, Yeon Hee Ryu

**Affiliations:** ^1^Nano Primo Research Center, Advanced Institute of Convergence Technology, Seoul National University, Suwon 443-270, Republic of Korea; ^2^Departments of Chemical Engineering, University of Louisville, Louisville, KY 40292, USA; ^3^Korea Institute of Oriental Medicine, Daejon 305-811, Republic of Korea

## Abstract

The primo vascular system (PVS) was first introduced by Bong-Han Kim via his five research reports. Among these the third report was most extensive and conclusive in terms of the PVS anatomy and physiology relating to the acupuncture meridians. His study results, unfortunately, were not reproduced by other scientists because he did not describe the materials and methods in detail. In 2002, a research team in Seoul National University reinitiated the PVS research, confirmed the existence of PVS in various organs, and discovered new characteristics of PVS. Two important examples are as follows: PVS was found in the adipose tissue and around cancer tissues. In parallel to these new findings, new methods for observing and identifying PVS were developed. Studies on the cell and material content inside the PVS, including the immune function cells and stem cells, are being progressed. In this review, Bong-Han Kim's study results in his third report are summarized, and the new results after him are briefly reviewed. In the last section, the obstacles in finding the PVS in the skin as an anatomical structure of acupuncture meridian are discussed.

## 1. Introduction: A Brief Historical Review

It was in 1962 when Bong-Han Kim (hereafter BH Kim or Kim) reported his first study results on the anatomical entity of acupuncture meridians (AM) [1]. He then established and became the director of National Acupuncture Meridian Research Institute in 1963 in Pyungyang, North Korea. The Institute produced four additional reports on this new system [2–5] until October 1965, when the institute abruptly closed for some unknown reasons. His fate after the event is still unknown.

The first report was very brief and mostly about the electric response of acupoints, which was probably not considered very exciting. The second report, however, contains the discovery of a completely new system, constituting node-like anatomical structures at the acupoints and the tube-like structure (Kim claimed it to be the AM) connected to the nodes in the skin [2]. The research team named the nodes the Bonghan corpuscles (currently renamed as primo nodes) and the tubes the Bonghan ducts (primo vessels). They also found that this new system existed not only in the skin but also throughout the body, including on the surfaces of body organs and inside the blood and lymph vessels. This new discovery became the foundation of the Bong-Han theory. To publicize its scientific achievements, the North Korean government translated Kim's second report in various languages including English and disseminated it to most major libraries in the world [6]. The third report was an extension of the second one, observing the entire network of the Bong-Han system (renamed as primo vascular system (PVS)) in the mammalian body [3]. The fourth one was about the “Sanal,” (renamed to be Primo-microcell (P-microcell)), whose functions, he claimed, were regeneration and/or repair, as totipotent stem cells [4]. Sanal is a Korean word and its direct translation in English is “live egg.” The last (fifth) one was a brief report about the hematopoietic function of the Sanal [5]. These five publications were reports rather than journal articles, and they described mainly results with insufficient information on methods and materials. The introduction and discussion sections were very short.

Shortly after Kim's fifth report, neither he nor his publications reappeared in public for some unknown reason, and his work was completely neglected by the North Korean government. Outside the North Korea (Democratic People's Republic of Korea; DPRK), there have been several attempts to reproduce Kim's results but without success, probably because details of his methods to identify this new organ were described either in any of his reports or elsewhere. Nevertheless, until now, there has been no known serious attempts to either confirm or negate his claims, with exceptions of the following two cases: one was by Kellner, who thoroughly investigated the acupoints in a histological manner but failed to find the structure that Kim had claimed [7]. In our scientific opinion, Kellner's conventional method for histological study was not appropriate for detecting the anticipated structure in the skin, because in the cross section of the tissue containing an acupoint this new organ may not be differentiated from its surrounding tissues. Optically and histologically, both look very similar if they are seen in their cross sections. Its presence can be revealed most likely in the longitudinal view, with application of appropriate dyes, according to our own experiences for the past ten years. Another case was by Fujiwara and Yu, who partially confirmed Kim's discovery but incompletely [8]. Fujiwara, who was an assistant professor in anatomy, then, later recalled that it took about a half year of hard work to get some positive results [9]. He was able to reproduce Kim's results inside blood vessels and on the surfaces of organs, which eventually helped the research of Soh, one of the coauthors for this paper.

Soh was a professor in the Department of Physics at the Seoul National University (SNU) during 1976–2011. He formed Biomedical Physics Laboratory in 2000, initially to investigate the biological phenomena related to the acupuncture therapy using physical means, such as electricity, magnetism, acoustics, and optics. He realized that his investigation without anatomical bases would not lead to the fundamental mechanism behind the acupuncture therapy. Therefore he formed a scientific team to investigate the Bong-Han theory with Dr. BC Lee as the main experimental partner. The SNU team soon successfully confirmed the PVS presence inside the blood vessel of rabbits. However, this initial success soon faced difficulties in reproducibility. They realized that the research requires highly skilful researcher in microsurgery and optical imaging due to the extremely small size and the semitransparent nature of the organ, which frequently impeded the progress in their study.

The team, therefore, decided to seek Professor Fujiwara's help and Soh visited Dr. Fujiwara in Osaka. Fujiwara himself had experiences with failures in finding the PVS on the surfaces of internal organs for more than six months. Fujiwara was so kind to provide a movie containing the experimental procedures that he had developed in 1960s. With Dr. Fujiwara's method, the SNU team then was able to reproduce his results. Since then, the team identified the PVS floating on the surfaces of intestines, liver, stomach, and bladder of rabbits. Soon, more thorough, histological and morphological studies on PVS were performed to confirm Kim's claims. With techniques and experiences obtained for the organ-surface PVS, the team moved forward to find the PVS floating inside lymph vessels. More importantly, a technique of using Trypan blue for PVS identification was developed by Dr. BC Lee, which enabled the team to identify the PVS in other organs, such as in the bovine heart, abdominal adipose tissues, brain ventricles, and the central canal of spinal cords. The Trypan blue technique also led to the discovery of the unique characteristics of the PVS in/on cancerous tumors (cancer PVS). This may probably be one of the most significant findings in the medical science because cancer is one of the most serious, life-threatening diseases. 

Until 2008, the SNU-team was the only one performing the PVS research and, therefore, the research progress was rather slow. In 2009, a review article, written by Soh, covering the PVS research progress during 2002 and 2008 was published in the Journal of Acupuncture Meridian Studies [10]. Since then several research teams in Korea participated in the PVS research, and the number of teams and their research subjects have been steadily growing. Outside Korea, the PVS gained interest as a research topic, mainly in China and USA. In September 2010, an international symposium on the PVS, The Primo Vascular System, Its Role in Cancer and Regeneration, was held in Korea, and its proceedings was published by the Springer Publishing Company in 2011 [11].

Recently, a research team led by BS Kwon at the National Cancer Center of Korea [12] confirmed that the PVS was abundant with immune cells such as macrophages and mast cells, which had been previously noticed by the SNU team [13]. In addition, primo nodes were also found to be packed with very small, embryonic stem cell- like cells. These data are consistent with the claims by BH Kim's on the properties of the PVS on regeneration and wound healing [4].

In the remaining part of the paper, we summarize the content of Kim's third report, which relates the AM system with the PVS, with our comments. His report contents are compared with the recent works reported by the SNU group and other PVS scientists. Kim's study results that were scientifically verified by the PVS researchers were described first, and then new discoveries on the PVS after Kim were introduced. The desired directions for the future PVS research were also discussed.

## 2. Acupuncture Meridian System

The title of Kim's third report is “The Kyungrak (*經絡*) System” [3], and it is officially submitted by the “Kyungrak Institute (*經絡* *研* *究* *院*) of Democratic People's Republic of Korea,” where Kim was the director. The English translation of the title is “Acupuncture Meridian System.” The report covers research results on the PVS network, and the scientific standard of its content is more advanced and comprehensive than that of his previous two reports. This was, in fact, his last report relating the acupuncture meridians with the PVS. The last two of his reports were about the “Sanals” or P-microcells [4, 5], and they were published shortly before the institute was abruptly closed in 1965.

The English translated table of content of the third report was presented in Box 1 for the readers to easily capture the breadth of Kim's work. As can be seen in the box, the third report covers not only anatomical and histological aspects of PVS but also its basic physiological aspects. The conclusion section of the report is in fact a good summary of the report, which was highly beneficial to future PVS researchers. We, therefore, translated this section (pages 36–38 of Kim's third report) with our own scientific comments, although the section by itself was already published in the book The Primo Vascular System [14]. We also added some of the important scientific progresses in the field of PVS, since Kim's publications, in subsequent sections.

### 2.1. English Translation of Conclusion Section of Bong-Han Kim's Third Report with Authors' Comments

#### 2.1.1. The Bong-Han System (BHS) Is Composed of Several Subsystems

 (A) These subsystems have common properties of possessing Bonghan ducts (BHDs) and Bonghan corpuscles (BHCs). All BHCs are interconnected via BHDs. BHDs connect BHCs. A BHD is composed of one to tens of Bonghan ductules.
    (1) A ductule has a thin layer, composed of endothelial cells with rod-shaped nuclei. It is surrounded by an external membrane (endo-BHD), which is made of smooth muscle-like cells and fine argentaffin fibers. (Comments: Kwon et al. observed epithelial cells rather than smooth muscle-like cells [12].) The interluminal space in a BHD is filled with fibrous and amorphous materials. These ductules are wrapped together with a membrane (peri-BHD) to form a single BHD. This outer membrane is made of membranous cells. In the lumen of a ductule, basophilic the granules and nucleus-like bodies are present.
    (2) The BHC is essentially formed by the enlargement, branching, or merging of the ductule. Also, the basic compositions of the BHC are the outer membrane of the ductules and the reticular fibers, extracellular matrices (ECM) between ductules. Inside the lumen of the BHD, which is extended from the BHC, basophilic granules, cell components, and chromaffin granules are present.
 (B) The BHS is classified as described in the following.
   (1) Intravascular BHS. This BHS class consists of the intravascular (IV) BHDs and BHCs. It is systematically distributed inside blood and lymphatic vessels along the vessel and inside the heart. (Comments: Up to now, the authors observed that the BHS is only in large caliber blood or lymphatic vessels. In the BH Kim's report, the size of the BHS-containing vessels is not clearly described.) The BHDs in this class are very fragile, and the ECM and their outer membranes (epi-BHD) are not well developed. The IV-BHC has a structure, particularly similar to hematopoietic organs. In the reticular BHS, lymphocyte series and myelocyte-like cells (cells in bone marrow) are present. Sometimes cells similar to organ parenchyma cells are gathered around.
   (2) Organ surface BHS. This class of the BHS consists of the organ surface (OS) BHDs and BHCs. They freely float on the surfaces of internal organs and are not associated with blood or lymphatic vessels. For this class BHDs, the interluminal materials and the outer membrane are developed better than those for the IV-BHS. In the lumens of the BHDs and inside BHCs, there are cells possessing bright cytoplasm as well as the basophilic granules.
    (3) Extravascular BHS. This BHS class made of the extravascular (EV) BHDs and BHCs. This runs along the blood and lymphatic vessels, and nerves. It is covered with thick connective tissues. In the lumens of the BHDs and inside the BHC, many chromaffin granules are present.
   (4) Nervous BHS. This class BHS is composed of the nervous (N) BHDs and BHCs, and it floats in the cerebrospinal fluid. Its branches are distributed in the parenchyma of the central nervous system and in the peripheral nervous system.
   (5) Intraorgan BHS. Inside the parenchyma of internal organs, there are intraorgan (IO) BHDs and BHCs, terminal BHDs, and terminal BHCs. (Comment: the terminal BHD has only a single lumen, and it is a type of the ductule.) These are extension of the BHDs originated from IV-, EV-, or N-BHDs and present inside of the organ. Individual BHDs merge together in an IO-BHC and eventually form terminal sub-BHDs. These individual terminal sub-BHDs are directly connected to each nucleus of the organ cells. Again, fine these fine ductules come out from these cells.
 (Comment: In summary, there are five subsystems, namely, IV-, OS-, EV-, N-, and IO-BHS). The BHS subclasses are well connected to each other. The IV-BHS is connected to the OS-BHS after coming out of the vessel wall. It is also connected to the EV-BHS via the EV-BHC. The OS-BHS is connected to the EV-BHS and the N-BHS. The communication among BHS subclasses is well established.

#### 2.1.2. The BHS Is Made of Multiple Systems Circulating Bonghan Liquor

 (A) Biochemical compositions of Bonghan liquor (i.e., liquid flowing inside the PVS) are
    (1) abundant in DNA and RNA;
    (2) total nitrogen content is 3.12–3.40%. Non protein nitrogen content is 0.10–0.17%. Lipid is 0.57–1.00%. Reduced sugar is 0.10–0.12%;
    (3) total hyaluronic acid is 170.4 mg%;
    (4) more than 19 free amino acids are present including several essential amino acids;
   (5) there are more than 16 free mono nucleotides.
 (B) The BHS possesses bioelectrical activities and mechanical motion.
    (1) The propagation speed of the electric response from the BHD is very low and is similar to the two types of waves (*ㄱ* and *ㄴ*) displayed by the BHC. The BHD responses to electric stimuli appear in various forms. (Comment: there were two types of wave forms that Kim found: *ㄱ* and *ㄴ*; these symbols are Korean alphabet letters.)
    (2) When a BHD is stimulated bioelectric signals propagate through the BHD. The speed of propagation is faster (1–3 mm/sec) for the waves with a smaller amplitude and slower for the greater.
    (3) The BHD has a spontaneous motion. This motion propagates and changes when the BHD is stimulated. Its longitudinal, oscillatory motion is either continuous or periodic. The transversal motion is vibratory. These suggest that the BHS is capable of actively circulating the Bonghan liquor.
 (C) All cells are connected to the BHS.
    (1) The nucleus of each cell has very small, entering and exiting terminal ductules. These ductules are connected to the BHCs in the internal organs. These IO-BHSs are connected to the cells only in their neighboring area. The IO-BHC is connected to the BHS of other classes. In other words, the BHS networks in various classes leave from and arrive at IO-BHSs.
 (D) The circulatory paths observed by the radioactive P^32^ injected into various points of the BHS are as follows.
   (1) The Bonghan liquor from all tissues circulates to the BHS in the skin. (Comment: The BNS in the skin may be the known acupoints and probably many unknown points.)
    (2) The Bonghan liquor flows from the BHS in the skin to the BHS deep inside the body.
   The fluid in the deep BHS flows to the IO-BHSs and then to the cells in the tissue. These data agree with the circulation study results obtained using dyes.
 (E) The BHS circulatory path is not a singular system.
   Unlike the blood circulation system, the Bonghan liquor circulatory path is not singular but multiple. These paths are connected but independent.
   A dye or isotope injected into a specific network circulates only in the region of its particular network.
   However, the Bonghan liquor in a particular path crosses to another one through the connecting routes between the two paths.

#### 2.1.3. A Change in the Bonghan Liquor Condition Affects the Functions of Organs

 (A) Stimulation on the BHD affects the pulsation frequency and amplitude of the heart and changes the peristaltic motion of intestines. It also affects the hysteric curves of skeletal muscles significantly.
 (B) Severing a BHD significantly affects the cells of the tissues connected to the BHD.
    (1) It causes the dissolution of the nuclei and consequently death of the cells.
    (2) If the BHD responsible for a peripheral nerve is severed, the excitability of the nerves reduces significantly.
    (3) If the BHD of a motor nerve is severed, for a certain period of time, the associated muscle does not show movement responding to the repetitive stimulations.

#### 2.1.4. In Terms of the Stage during the Differentiation and Development. The BHS Precede Those of Blood Vessels, Nerves, and Other Organs

The typical developmental stages of BHD, for chicken egg, from incubation are: the 7–8th hours, BHD blast; the 10th hours, pre-BHD; the 15th hours, proto-BHD; and the 20–28th hours, fully developed BHD. The earlier timeline of the BHS differentiation and development suggests its roles in the development. (Comments: in the report, there is no description on its roles.)

#### 2.1.5. BHS Exists Broadly, in All Levels of Life

The BHS is proven to exist not only in the mammalian but also in all vertebrates and invertebrates. It exists even in the plant. We conjecture that it exists in any multicellular lives

The study results on the BHS suggest that the BHS circulation rout is cells in the tissue → skin BHCs → deep BHCs → intraorgan BHCs → terminal BHSs → cells in the tissue.

The BHS is made of multiple, independent circulating systems (subsystems), which are interconnected each other, but forming a single coherent system.

All organs of the living beings are connected to and controlled by the BHS. In other words, all forms of lives have their own BHS. 

## 3. Confirmation of BH Kim's Study Results

The nature and the scale of BH Kim's accomplishments on the PVS are enormous. Since 2002, selected parts of BH Kim's studies were repeated, and his results were confirmed to be accurate. This section summarizes BH Kim's findings that were verified fifty years later, by various scientists. Here, we briefly list the original terms and the new ones of the system, for your convenience. Bong-Han system (BHS) = primo vascular system (PVS), Bonghan duct (BHD) = primo vessel (PV), Bonghan corpuscle (BHC) = primo node (PN), Bonghan ductule = P-subvessel, Bonghan liquor = primo fluid (P-fluid), and Sanal = p-microcell.

### 3.1. Animal Species and Organs Studied

Animal species studied for identifying the PVS were mainly rabbits, rats, and mice. For a few cases, pigs, dogs, cows, and human placentas were also studied. Among the PVS subclasses, defined by Kim (Box 1), the IV-, OS-, and N-PVS were confirmed partially, if not completely.

 The IV-PVS floating inside blood vessels was first identified in the abdominal artery and the *caudal vena cava* of rabbits [14], rats [15], and mice [16]. More importantly, the PVS in the atrium of a bovine heart was found to form a floating network [17]. The PVS in the sagittal sinus of a rat brain was recently identified [18].

 The IV-PVS floating inside lymph vessels, was visualized with help of Janus Green B [19], fluorescent nanoparticles [20], Alcian blue [21] and with no contrast agent [22]. The morphological information on PVS [23] and protocols used for PVS related experiments [24] were recently published. The fact that the PNs possess large amount of cells and granules related the immune system may imply that the PVS is involved in protecting function of the body [12].

 The OS-PVS floating on the surfaces of internal organs was observed in rabbits [25] rats [26], mice [27], dogs [28], and pigs [29]. The structure of this PVS subclass was characterized by the optical [25], and electron microscopy [13, 30].

 The N-PVS floating in the cerebrospinal fluid (CSF) in the brain of a rabbit [31] and a rat [32] was optically observed, by use of Trypan blue. The PVS in the subarachnoidal space of the rabbit [33] and rat [34] brains, and in the spine of the rat [35] and pig [36] were also identified.

All PVS observed and listed previously were floating in the body fluid, such as blood, lymph, abdominal fluid, and CSF.

### 3.2. Brief Summary of Important PVS Characteristics Verified

#### 3.2.1. Confirmation of PV Features

**Figure 1:**
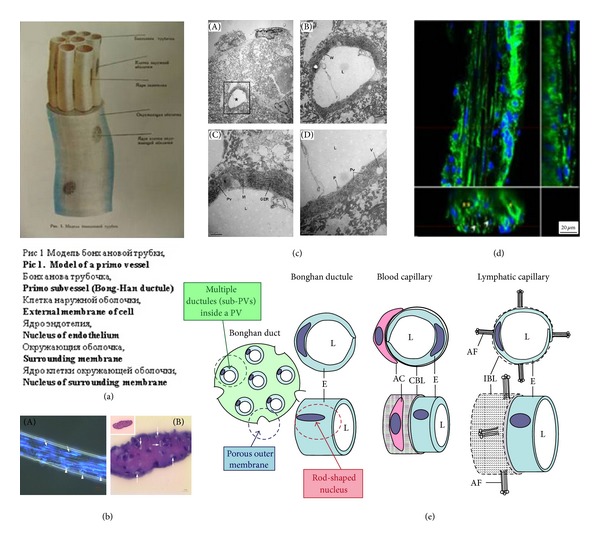
PV (Bonghan duct) illustrated in various publications. (a) Schematic diagram of a primo vessel, described by Bong-Han Kim with Russian terminologies. English translation of the terminology is added in bold [3]. (b) Optical images showing histological characteristics of a PV. (A) Phase-contrast image of a PV with DAPI. Several sub-PVs (arrow) with rod-shaped nuclei (light blue, arrowhead) are seen. (B) The cross section of a PV: several lumens (arrows) are seen [37]. (c) Electron microscopy of a partial cross section of a PV. (A) A lumen (asterisk) of a sub-PV can be seen. (B) Magnified image of the lumen (rectangular area in (A)). The wall (W) of the sub-PV consists of a single layer of endothelial cells surrounded by fibrin-like fibers. ((C), (D)) Magnified image of (B). L: lumen; M: mitochondria; GER: granular endoplasmic reticulum; P: cytoplasmic protrusion; PV: pinocytotic vesicles; V: vacuole; and F: fibrin-like fiber [37]. (d) Confocal laser scanning microscope image of a PV. The main panel is optical microscopy of the longitudinal section of a PV (the middle section, cells with rod-shaped nuclei in blue), accompanied by a venule and an arteriole on each side (cells with circular nuclei). The lower panel is a cross section of the PV, showing multiple lumens (open arrowheads), and the venule (asterisk) and the arteriole (two asterisks) on its each side. The PV diameter was approximately 30 *μ*m [33]. (e) Anatomical comparisons of characteristics of the PV, blood, and lymphatic capillaries. The PV has multiple sub-PVs within, and the PV's outermost layer has large pores. The endothelial cells of the sub-PV possess rod-shaped nuclei. E: endothelium; L: lumen; AC: accessory cell; CBL: complete basal lamina; IBL: incomplete basal lamina; and AF: anchoring filaments [37].

One of the most distinguished anatomical features of the PV is its bundle-like structure made of multiple subvessels.  Figure 1(a) shows a schematic diagram of a PV prepared by Kim [3]. Throughout BH Kim's five reports, he, however, did not provide any actual image or the method to observe the PVS, corresponding to the diagram. It has been, therefore, difficult to obtain the PV image with its cross section. The image published in 2009, with hematoxylin and eosine staining, is shown in Figure 1(b) [37]. The existence of P-subvessel lumens is indeed shown in the figure, but the shape of lumens is still not clearly shown. Thanks to better techniques in the sample preparation and microscopy, imaging of a PV cross section, without severe deformation, is now possible. In one of most recent study results with an OS-PVS, a PV cross section imaged utilizing transmission electron microscopy (TEM; Figure 1(c)) [37] shows even the endothelial cell layer of a PV. The surrounding ECM is made of collagen fibers, as BH Kim claimed 3.  Figure 1(d) shows confocal microscopy images of the longitudinal and cross sections of a PV sample harvested from the superior sagittal sinus of a rabbit brain [33], which clearly shows multiple lumens in a PV. We, therefore, suspect that the difficulties in appropriately preparing PV samples and in having good microscopy techniques might be the reason why BH Kim did not provide images of PV cross sections.  Figure 1(e) is a schematic illustration of the PV compared with blood and lymphatic vessels displayed by Ogay et al., which summarizes most distinctive anatomical features of the PVS [37].

#### 3.2.2. Cells inside the Primo Node

**Figure 2:**
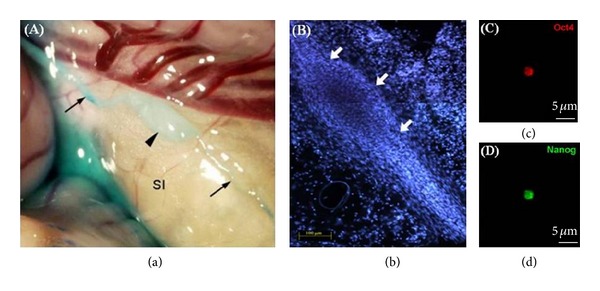
Images of PVS. (a) An image of a PN (Bonghan corpuscle; arrowhead) found on the rabbit small intestine, with PVs (arrows) at both ends, using methylene blue as the contrast agent [37]. (b) An image of a PN (arrows), which was identified lower part of the superior sagittal sinus of a rabbit brain [33]. DAPI staining of the nuclei of the cells inside the PN. Very small cells are packed in the PN. ((c), (d)) Immunostaining of the small cells isolated from PNs on the surfaces of rat intestine, for the embryonic stem cell markers Oct4 (red) and Nanog (green). The scale bar indicates 5 *μ*m [38].

The PN is surrounded by a thin membrane, usually connected to two or more PVs (Figure 2(a)), and contains many cells (Figure 2(b)) and embryonic stem cell-like bodies (Figure 2(c)). Recent studies on PN have been mainly on the P-microcells (or Sanals) and other types of cells in it. Presence of many cells involved in immune functions was first noticed by TEM in the PN sample obtained from the rabbit organ surfaces [13]. The cell types and the ratio of the cells in the PNs harvested from the internal organ surfaces and inside the lymphatic vessels of rats are [12] mast cells (20%), eosinophils (16%), neutrophils (5%), histiocytes (53%), lymphocytes (1%), and round immature stem cells (3%). Although the presence of immune cells in PNs was not specifically mentioned in BH Kim's report, he did emphasize the abundance of chromaffin cells [3], which was confirmed by Kwon et al. [12].

The presence of BH Kim's Sanals (P-microcells) in the PVS was studied by the SNU team from its inception [25]. The cells were found to show a peculiar motion to the light in the UV-A range (360 nm) [40, 41]. Later, the budding process for cell proliferation was identified by atomic force microscopy [42–44]. An implication for these cells to be embryonic-like stem cells were made after confirming the expression of the stem cell biomarkers oct4, nanog, and CD133 [12, 38]. More studies on the Sanal are being in progress.

#### 3.2.3. The Primo Fluid and Its Flow Direction

BH Kim's study results on the primo fluid circulation were confirmed only in a very limited level. The flow of the primo fluid in a certain path was demonstrated in the study using Alcian blue, from the rat acupoint BL-23 in the dorsal skin to the PVS on the surface of internal organs [45]. The experiment was, however, not always repeatable, and the reason for this inconsistency is still unknown.

When Chrome-hematoxylin and fluorescent nanoparticles were injected into testis they were found in the PVs on the organ surfaces between the abdominal cavity and the abdominal wall, although farther tracing was not technically possible with optical microscopy [46]. Tracing primo fluid in the PV on rabbit organ surfaces was possible using Alcian blue, and the flow speed was measured to be 0.3 ± 0.1 mm/sec [30]. This value was consistent with the values in BH Kim's reports [3].

One of the important biochemicals in the primo fluid, which was mentioned in BH Kim's report, was catecholamine (adrenalin and noradrenalin) [3], and its presence was later confirmed in the PNs on the organ surface of rabbits [47] and rats [12], using ELISA.

## 4. New Discoveries

Since the initiation of the PVS research, the SNU team made significant discoveries on PVS as well as developing new techniques for identifying PVS in the mammalian body. A few important and medically relevant findings are presented here.

### 4.1. The PVS in Adipose Tissues

**Figure 3:**
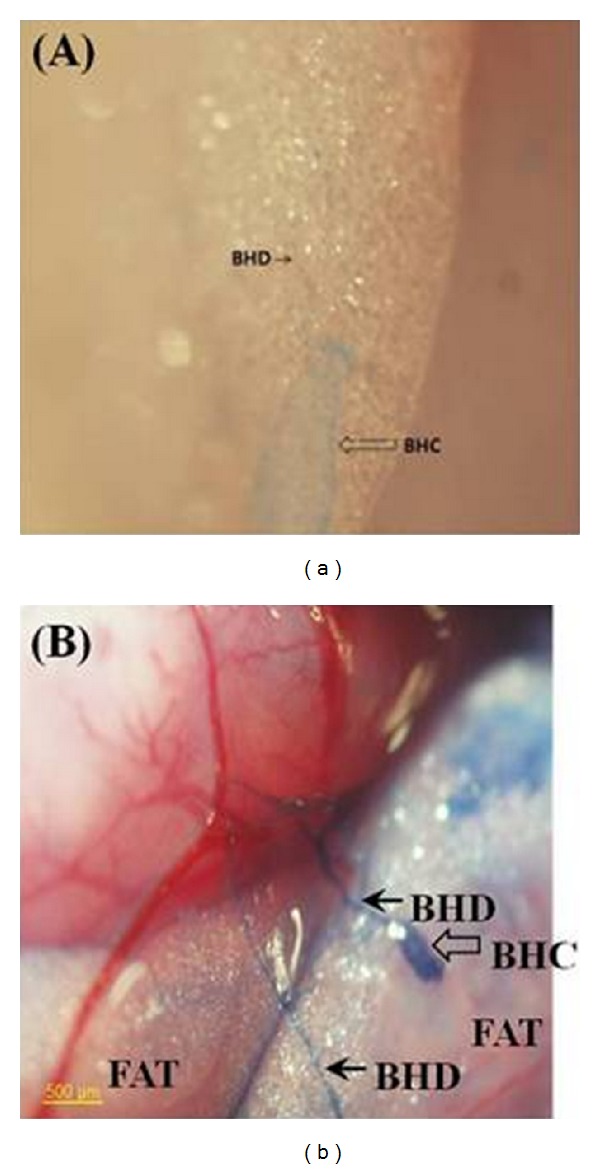
Images of PNs with different dyes. (a) A PN (BHC) and a PV (BHD) connected to the PN, on the adipose tissue around the rat small intestine. Alcian blue flow through the PVS left it pale blue. Notice that Alcian blue did not remain in the PVS and, therefore, *in situ* tracking of the PVS using this dye was difficult. (b) A PN and PVs, observed near the rat small intestine, stained with Trypan blue. Notice that the blood vessel and adipose tissue were not stained [39].

The presence of the PVS entering the adipose tissues around the rat small intestine was first noticed via optical imaging using Alcian blue, which was injected intravenously at the femoral vein. The Alcian blue entering a PVS floating inside a blood vessel reached to a PN in an adipose tissue as shown in Figure 3(a). Unfortunately, the dye flowed away without showing its previous paths. Therefore, *in situ* tracking of the PVS using this dye was found to be inappropriate [39].

Trypan blue, an alternative, was, therefore, tested, and it remained inside the PVS, allowing us to visualize the system in the adipose tissue. Trypan blue was found to stain the PVS preferentially to the adipose tissues or blood vessels [39].  Figure 3(b) illustrates images of the PVS, using Trypan blue, in the adipose tissues around the small intestine of a rat. The presence of PVS in adipose tissues raises conjectures on its possible roles in connection with regeneration, obesity, and obesity-related diseases. 

### 4.2. Cancer PVS

Existence of PVS on the surface of the tumor membranes in a xenografted mouse was first observed by Yoo et al. [48] of the SNU team. It was soon realized that PVS was more densely populated in proximity of tumors (xenografts) in various type of cancerous, human origin tumors, and, therefore, they were easily identified [27]. More importantly, significantly large number of cancer cells was found in PVs connecting the primary to the secondary tumors [49].

Since these initial findings by the SNU team, research teams of Akers [50], Hong [51], Heo [52], Islam [53], and Kang [54] confirmed the presence of high density PVS (cancer PVS) in close proximity of cancer xenografts in mice. A PV floating inside a lymph vessel originated from the tumor xenografted in the abdominal skin of a mouse was also reported [55].

The Miller team also reported that the cells obtained from the cancer-PVS of murine xenograft of human originated lymphoma U937 expressed CD68, CD45, and lysozome. They also revealed that the immunophenotype of cells inside the cancer PVS is of U937 cell. The cells also showed hundreds to thousandsfold, upregulated KLF4, one of the human cancer stem cell specific transcription factors and an upstream regulator of NANOG, which maintain the pluripotent and undifferentiated state of stem cells [53].

### 4.3. Other New Findings

For the PVS studies, the SNU team has been using traditional histochemistry techniques with dyes such as Alcian blue, Trypan blue, chrome-hematoxylin. Among these the Trypan blue spraying technique invented by Dr. BC Lee was most effective for many applications. The team also adopted modern imaging techniques utilizing fluorescent nanoparticles, quantum dots, immune-affinity technique, electron microscopy [56], X-ray microscopy, and GFP expressing cells and animals, which were not available at the time of BH Kim. DAPI staining to check the shape of cell nuclei is one of the important and frequently used new techniques for the PV identification.

 In Kim's reports, only white blood cells among cells and biochemicals are involved in the immune system. The concept of the stem cell, especially of the adult stem cell, was not very well known in his time, but he claimed that P-microcell (Sanal) is the main agent for wound healing and regeneration, which are the two fundamental roles of stem cells [4]. Considering that the Sanal size is very small (1–5 *μ*m), an important question is the relation between the Sanal and the very small embryonic-like stem cells (VSEL) described by Ratajczak et al. [57], because both seem to have very similar characteristics. The budding of Sanals was previously observed by atomic force microscopy [43], and the expressions of several stem cell biomarkers on/in Sanals are confirmed. Detailed and various aspects on the Sanal should be investigated because the important nature of this particular cell. Kim also claimed that the proliferation of Sanals was affected by the light, which has not been confirmed yet, although the average movement activities of the Sanal in liquid was found to increase when the light at 360 nm (UV-A) was illuminated [40]. 

PVS does not express CD31 (blood vessel specific marker) and LYVE-1 (lymphatic vessel biomarker), confirming that the PVS is different from the blood or lymphatic systems. Proteomics study results on the PV and P-fluid harvested from the rabbit organ surface [58] revealed that keratin 10 was present in the PVS. Results of western blot [59] and immunohistochemistry [12] revealed that the keratin 10 was found to be from the epithelial cells of the PVS outer surface. Epithelial marker protein 3 (EMP-3) [12] was also found in the outer membrane of PVS, and von Willebrand Factor (vWF) was present in the PVS endothelial cells. 

## 5. Concluding Remarks

Fifty years ago, BH Kim showed the relationship between the acupuncture meridian and the PVS by injecting a blue dye into an acupuncture point and by observing the dye flowing via the meridian, and concluded that the meridian system belongs to the Bong-Han system (PVS). Until now, the blue dye that BH Kim used is not known, and most of his claims on the meridian and the Bong-Han system are still to be verified, although many of his study results that could be repeated until now are found to be very important for the modern biomedical sciences. Approximately fifty years after BH Kim, the presence of the PVS inside the blood and lymphatic vessels, cerebrospinal fluids of the central nervous system, and on the surface of the various internal organs was indeed confirmed by various techniques developed by the SNU team [60]. The most significant unconfirmed part is ironically the PVS in skin, which is supposed to be the acupoint. BH Kim claimed that the acupuncture meridians extended into the PVS inside the mammalian body, which still needs to be verified because the techniques used for the PVS inside the body do not appear to work for the PVS in the skin.

 A proposed detection procedure for identifying the PVS in the skin is as follows. (1) First, perform proteomics and genomics of the PVS with the PVS specimens that can be now harvested, and identify PVS specific biomarkers. (2) Then, develop these biomarker-specific, targeting biomolecules, such as antibodies or aptamers. (3) Apply the appropriate image contrast agents conjugated-targeting molecules into PNs. (4) Trace the contrast agents in appropriate image modalities to map the PVS in the entire body [61]. In this way, the entire PVS network, including the ones in the skin (acupoints), is expected to be visualized.

Currently, the main obstacles to this proposed approach are in the difficulties in obtaining sufficient quantity of pure PVS samples using the current techniques, due to the very small size of the PVS, and in identifying proper imaging modalities that can provide both sufficient sensitivity and resolution. Nevertheless once the PVS-specific biomarkers are identified, the rest is expected to be resolved with less difficulties.

We strongly believe that the thousand-year-old acupuncture therapy and traditional eastern medicine will become a true sense of the scientific medicine when the entire network of PVS and its roles in mammalian body are fully uncovered. This will then shift the level of the oriental medicine from the traditional wisdom and art with a long history to the biomedical sciences in true sense. Furthermore, it will also bring a paradigm change in the regenerative medicine, cancer, immune deficiency or hyperactivity, pain control, stem cell therapy, and other important issues in the human health care in general.

## Figures and Tables

**Box 1:**
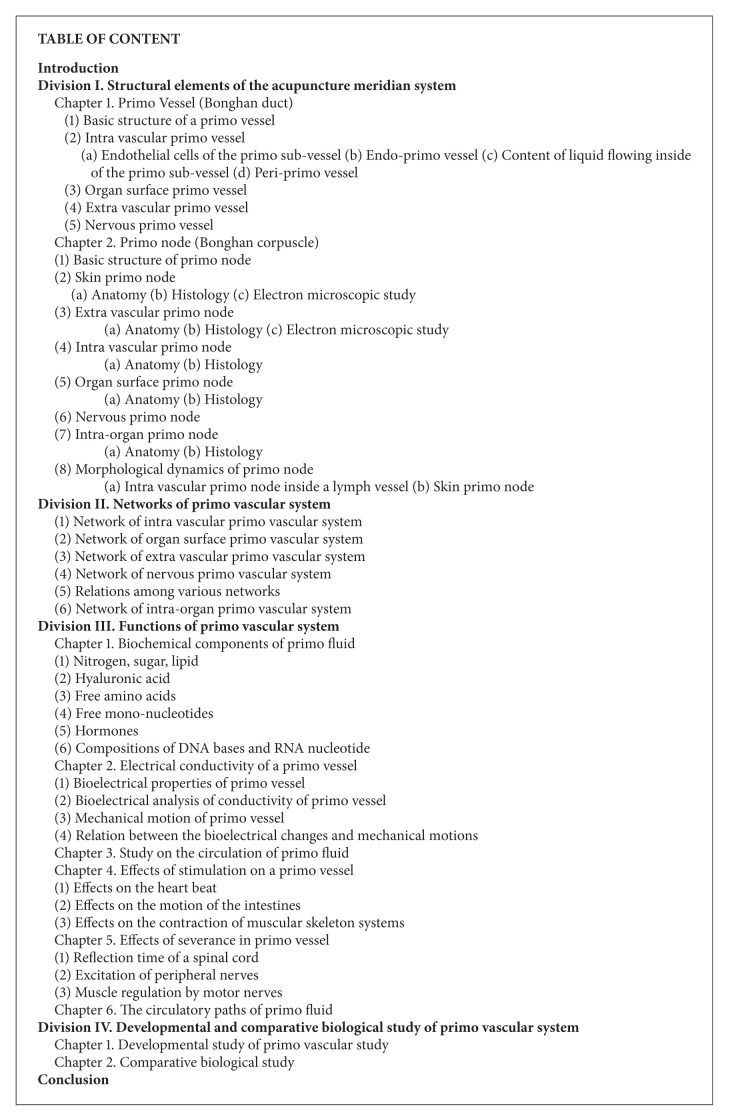
Content of Bong-Han Kim's third report, (it should be noted that, in the box, the terms related to the Bong-Han System are translated into the terms of Primo Vascular System).
